# Identification of a new *Clostridium perfringens* variant with a chromosomally encoded enterotoxin gene in a suspected persistent food poisoning outbreak in Eritrea

**DOI:** 10.3389/fmicb.2024.1459840

**Published:** 2024-10-15

**Authors:** Päivi Lahti, Kaisa Jaakkola, Ari Hörman, Annamari Heikinheimo, Ava Sovijärvi, Hannu Korkeala

**Affiliations:** ^1^Department of Food Hygiene and Environmental Health, University of Helsinki, Helsinki, Finland; ^2^Medical Services, Defence Command, The Finnish Defence Forces, Helsinki, Finland; ^3^Microbiology Unit, Finnish Food Authority, Seinäjoki, Finland; ^4^Retired, Turku, Finland

**Keywords:** *Clostridium perfringens*, genotyping, variant strains, enterotoxin gene, food poisoning, persistent outbreak, IS element

## Abstract

*Clostridium perfringens* is a causative agent of various human and animal enteric diseases including food poisoning. In this study, we describe an interesting case of a persistent food poisoning outbreak among Finnish peacekeepers in Eritrea, possibly caused by *Clostridium perfringens* carrying a new variant of the chromosomally encoded enterotoxin gene. *C. perfringens* strains causing food poisoning carry the enterotoxin gene, *cpe*, in its chromosome (c-*cpe*) or on a plasmid (p-*cpe*). PCR assays are widely used for toxinotype *C. perfringens* strains. The integration sites for the *cpe* gene are highly conserved, and PCR assays targeting the *cpe* gene and the adjacent IS elements (the IS*1470* in c-*cpe* and the IS*1470*-like or IS*1151* in p-*cpe* strains) are used to further determine the genetic location of the *cpe* gene. We sequenced nine enteropathogenic *C. perfringens* strains related to a persistent food poisoning outbreak among Finnish peacekeepers in Eritrea. Six of these strains produced non-typeable *cpe* results in the standard PCR assay due to divergence in the enterotoxin integration site. The gene order of the new variant of the chromosomal *cpe* insertion site with an additional IS1470 element impairing genotyping PCR assay for the location of *cpe* is described. In addition, variant c-*cpe* strains carried 58–81 copies of IS1470 in their genomes, compared to 9–23 copies found in previously described c-*cpe* strains. Thus, the present study represents an untraditional type of *C. perfringens* food poisoning caused by variant c-*cpe* strains, and the sequenced strains bring geographic variation to the existing strain collection of sequenced *C. perfringens.*

## 1 Introduction

*Clostridium perfringens* is an anaerobic, spore former causing gas gangrene, wound infections, and a variety of human and animal diseases involving the gastrointestinal (GI) system (Lindström et al., 2011; Kiu and Hall, 2018). Traditional *C. perfringens* food poisoning outbreaks are associated with temperature-abused food and typically affect many people at the same time. In addition, cases of antibiotic-associated diarrhea (AAD) by *C. perfringens* may be transmitted through food (Lindström et al., 2011).

Both food poisoning and AAD cases of *C. perfringens* diarrhea are primarily caused by type F strains (strains carrying enterotoxin gene *cpe*), which produce pore-forming *C. perfringens* enterotoxin (CPE). The prevailing understanding is that all *C. perfringens* strains may carry the *cpe* gene, but only approximately 5% do and produce CPE (Miyamoto et al., 2006). The *cpe* allele encoding a 319 aa end product is located either in pCPF5603 or pCPF4969 plasmid (plasmid-mediated *cpe,* p-*cpe* strains) or in a transposable element Tn5565 integrated into the chromosome (chromosomal *cpe*, c-*cpe* strains) (Cornillot et al., 1995; Brynestad et al., 1997; Miyamoto et al., 2006; Li et al., 2010). The Tn5565 includes insertion sequences (IS) IS1470 and IS1469 directly adjacent to the *cpe* gene (Brynestad et al., 1997). Known *cpe*-carrying plasmids are all conjugative and horizontally transferable (Miyamoto et al., 2006). The plasmid *cpe* gene is adjacent to IS1151- or IS1470-like elements (Daube et al., 1993; Miyamoto et al., 2002).

Foodborne outbreaks are caused by both p-*cpe* and c-*cpe* strains (Lahti et al., 2008), but multiple studies have shown that most outbreaks are caused by chromosomal *cpe* strains. In addition, a 325 aa variant of the *cpe* gene of unknown clinical relevance has been described in the pCPBB1 plasmid (Miyamoto et al., 2011). Persistent longitudinal outbreaks of *C. perfringens* have been suggested in some studies (Kiu et al., 2019), and these outbreaks have been associated with plasmidial *cpe*-carrying strains.

Six toxin genes detected by PCR have been widely used for toxinotype strains of types A–G (Rood et al., 2018). The only invariably chromosomal gene in the toxinotyping scheme is alpha (*plc*), which is present in all *C. perfringens* strains, while the other toxinotyping toxins are carried on transposable elements or a family of conjugative plasmids (Li et al., 2013). Toxinotyping, therefore, does not reflect the phylogenetic lineage of strains. To address this, the availability of genomic sequences has led to the establishment of genetic lineages (Kiu et al., 2017; Feng et al., 2020; Jaakkola et al., 2021), and a virulence gene profile scheme including chromosomal genes has been proposed (Abdelrahim et al., 2019).

PCR genotyping is also used to detect the location and type of enterotoxin gene in enteropathogenic *C. perfringens*. Despite the transmissible nature of these genetic structures, the gene order on both chromosomal and plasmidial integration sites is so highly conserved that IS elements are utilized as PCR probe targets (Brynestad, 1997), and reported variant strains have been related to the presence of a larger variant of the enterotoxin gene itself (325 aa) (Heikinheimo et al., 2006; Li et al., 2007).

In this study, we describe a persistent food poisoning outbreak putatively caused by Eritrean *C. perfringens* strains. In addition, we describe six outbreak strains with chromosomal *cpe* that exhibit an additional IS1470 insertion sequence near the *cpe* gene, which affects the ability of the PCR assay to detect the *cpe* location and genotyping results.

## 2 Materials and methods

### 2.1 Outbreak and stool samples

The occurrence of gastroenteritis among Finnish peacekeepers deployed in the United Nations peacekeeping operation in Eritrea in Africa (UNMEE) increased during the summer and autumn of 2004. In the autumn of 2004, an investigation team from Finland was sent to Eritrea to investigate the possible outbreak, take measures, and give instructions to address the situation. Inspections of the camp food premises, water distribution system, and local water suppliers were conducted. Water samples were collected and analyzed for the total bacterial count, total coliforms, *E. coli*, parasites (*Giardia, Cryptosporidium,* and *Entamoeba histolytica*), and noroviruses using the standard drinking water methods. No *E. coli*, parasites, or noroviruses were detected in any of the 13 samples.

Of the 184 peacekeepers, 163 (91.3%) answered the epidemiological questionnaire and 98 (60.1%) of them had suffered from symptoms such as diarrhea (93.9%), flatulence (69.4%), abdominal pain (58.2%), nausea without vomiting (51.5%), lack of appetite (43.9%), abdominal distention (33.7%), fever (31.6%), vomiting (22.4%), muscular pain (17.3%), abdominal cramps and other symptoms (16.3%), and bloody diarrhea (7.1%). The illnesses occurred consistently from June to October, with no distinct peak in their occurrence. The analysis of questionnaires and medical reports revealed that on average, approximately five peacekeepers were sick each day from June to October.

Stool specimens were collected from 184 Finnish peacekeepers (sample numbers 1–184) and 38 local Eritrean staff (cooks and cleaners) members (sample numbers 200–237) and transported to the Laboratory of Helsinki University Central Hospital. *Salmonella*, *Shigella*, *Yersinia*, and *Campylobacter*, parasites (*Cryptosporidium*, *Cyclospora*, *Cystoisospora,* and *Dientamoeba*), norovirus and astrovirus, and antigens for *Giardia*, *Cryptosporidium*, and *Entamoeba histolytica* were investigated from the stool samples using standardized methods of the laboratory. The epidemiological and laboratory investigations revealed no specific bacteria, virus, or parasite connected to the illnesses. However, the symptoms resembled those of *Clostridium perfringens* food poisoning, and the fecal samples were taken for further examination.

### 2.2 Detection and isolation of *Clostridium perfringens*

Each fecal swab sample was dissolved into a tube that contained sterilized water, and the hydrophobic grid membrane filter-colony hybridization (HGMF-CH) (Heikinheimo et al., 2004) was used to detect and isolate *cpe*-carrying *C. perfringens* from the samples. The samples giving a positive signal in the HGMF-CH assay were considered positive for *cpe*-carrying *C. perfringens*. The probe-positive colonies were isolated from each sample to obtain *cpe*-carrying *C. perfringens* from a single sample and to further study the genetic relatedness of these isolates.

### 2.3 PCR and PFGE typing

We detected major toxins and *cpe* in the *C. perfringens* isolates with PCR, as previously described by Heikinheimo and Korkeala (2005) in 2004–2005. The current nomenclature was used in this article (Rood et al., 2018). *C. perfringens* strains NCTC 8239, ATCC 3626, CCUG 2036, CCUG 2037, and CCUG 44727 were used as positive controls. *C. perfringens* type F isolates were further studied by PCR to determine the *cpe* genotype based on the *cpe* insertion site with IS elements. The total DNA was isolated by using Advamax beads (Edge Biosystems, Gaithersburg, MD, USA), according to the manufacturer’s instructions. IS elements downstream of *cpe* that determine the *cpe* genotype (IS1151, IS1470-like, or IS1470) of each isolate were characterized by using PCR with the previously described primers (Daube et al., 1993; Brynestad, 1997; Brynestad et al., 1997; Miyamoto et al., 2002; Miyamoto et al., 2004) and protocols (Heikinheimo et al., 2006).

In pulsed-field gel electrophoresis (PFGE) analysis, the DNA was digested with ApaI (New England Biolabs, Beverly, MA, USA), and the genetic relationships between isolates were assessed using the previously described assay (Ridell et al., 1998), which was modified by adding thiourea to the electrophoresis running buffer (Leclair et al., 2006). The digital images of PFGE patterns were analyzed using Bionumerics software (version 4.6, Applied Maths, Sint-Martens-Latem, Belgium), and the similarity analysis of PFGE patterns was performed using the Dice coefficient (optimized 2%, tolerance 1.2%). Clustering and construction of dendrograms were performed by using the unweighted pair-group method with arithmetic averages.

### 2.4 Sequencing of selected *Clostridium perfringens* isolates and annotation

Nine strains were sequenced in 2022 and have been deposited in the GenBank. These nine strains were selected for sequencing based on the genotyping results, and they represented both chromosomal and variant chromosomal *cpe-*carrying isolates in the genotyping assay.

Genomic DNA of *C. perfringens* isolates was extracted, as described by Keto-Timonen et al. (2006), and whole-genome sequencing was performed using PacBio RSII (Institute of Biotechnology, Helsinki, Finland). Sequenced genomes were assembled using HGAP3 and checked for circularity using Gap4 (Staden et al., 2003; Chin et al., 2013). To improve the draft assembly, Illumina MiSeq reads and the Pilon tool were used for genome polishing (Walker et al., 2014). Both sequenced and downloaded genomes were annotated using Prokka (Seemann, 2014).

Selected reference strains (ATCC 13124, SM101, and Str. 13) and previously sequenced lineage IV strains were included in the cgMLST analysis (Supplementary Table S1). Genomes were downloaded from the Bacterial and Viral Bioinformatics Resource Center.^1^

### 2.5 Comparative genome analysis

Bacterial and Viral Bioinformatics Resource Center (see text footnote 1) was used to perform comparative genome analysis and to combine results with previous studies (Jaakkola et al., 2021). To determine the cgMLST target gene set and create a genome-wide gene-by-gene comparison, ChewBBACA (3.0.0) was used (Silva et al., 2018). A schema created by Abdel-Glil et al. (2021) available at https://www.cgmlst.org was used. The core genome of 63 *C. perfringens* genomes consisted of 1,236 genes. For cgMLST results, a minimal spanning tree was calculated by GrapeTree (Zhou et al., 2018) using MSTreeV2. Strains (*n* = 63) included in the comparative genome analysis are listed in Supplementary Table S1.

Sequenced genomes were queried for the presence of IS elements (IS1470, IS1469, and IS1151) and selected genetic features (Supplementary Table S2). For IS elements, the hits with >80% identity over 80% of length were considered a match. For other genetic features, an identity threshold of 90% was used.

### 2.6 Phylogenetic analysis

Single-nucleotide polymorphisms between sequenced strains and selected reference strain genomes were identified using Snippy 4.6. (Seemann et al., 2015), with strain SM101 (289380.15) as a reference. A phylogeny based on core-SNP alignment was created by IQ-TREE 2.3.0. (Nguyen et al., 2015). Bootstrap values for branches were approximated using ultrafast bootstrapping (-B 1000), and FigTree v1.4.4. was used to visualize the trees (Rambaut and Drummond, 2008). Strains (*n* = 63) included in phylogenetic analysis are listed in Supplementary Table S1.

## 3 Results

### 3.1 *cpe*-positive *Clostridium perfringens* samples

Altogether, 50 of 222 (22.5%) human stool samples gave a positive signal in the HGMF-CH and were considered positive for the presence of *cpe*-carrying *C. perfringens*. The prevalence of type F *C. perfringens* differed between the samples from peacekeepers and local staff members. Overall, 32 of 184 (17.4%) of the samples of the peacekeepers and 18 of 38 (47.3%) of the samples of local staff members were positive. Altogether, we isolated 96 *C. perfringens* isolates from 12 samples (Table 1). The isolates were regarded as *cpe*-positive since they gave a signal in the HGMF-CH.

**Table 1:** *cpe*-positive samples yielded *cpe*-positive isolates, the isolation source of the samples, the number of *cpe-*positive isolates, and their *cpe* type (chromosomal/plasmidial).

Sample number	Isolation source	Number of *cpe*-positive isolates	Number of isolated *cpe* genotypes		
			c-*cpe* isolates IS1470	p-*cpe* isolates IS1470-like or IS1151	Variant isolates^a^
24	Peacekeeper	7	2	0	5
33	Peacekeeper	7 (+2 *cpe*-negative)	7	0	0
44	Peacekeeper	3	3	0	0
53	Peacekeeper	9	9	0	0
96	Peacekeeper	2	0	0	2
103	Peacekeeper	14	14	0	0
203	Local cook	5	4	0	1
204	Local cleaner	1	1	0	0
205	Local cleaner	32	2	0	30
206	Local cook	1	1	0	0
225	Local cook	3	3	0	0
233	Local cook	10	3	0	7

a Divergent size product in PCR detecting c-*cpe*.

Of the peacekeepers who gave *cpe*-positive or *cpe*-negative samples, 72 and 59%, respectively, had symptoms of intestinal disease between June and October 2004.

### 3.2 Genotyping results

Based on multiplex PCR, 94 (98%) of the 96 isolates were *cpe*-carrying type F (former type A). None of the 94 *cpe*-carrying isolates carried plasmidial *cpe*. PCR genotyping identified 49 (52%) of the 94 isolates as typical c-*cpe* isolates. The remaining 45 (48%) of the 94 isolates yielded PCR products of divergent size compared to c-*cpe*; thus, the genetic location of the *cpe* gene of these 45 isolates was interpreted as a variant. The PCR results are shown in Table 1.

**Figure 1:**
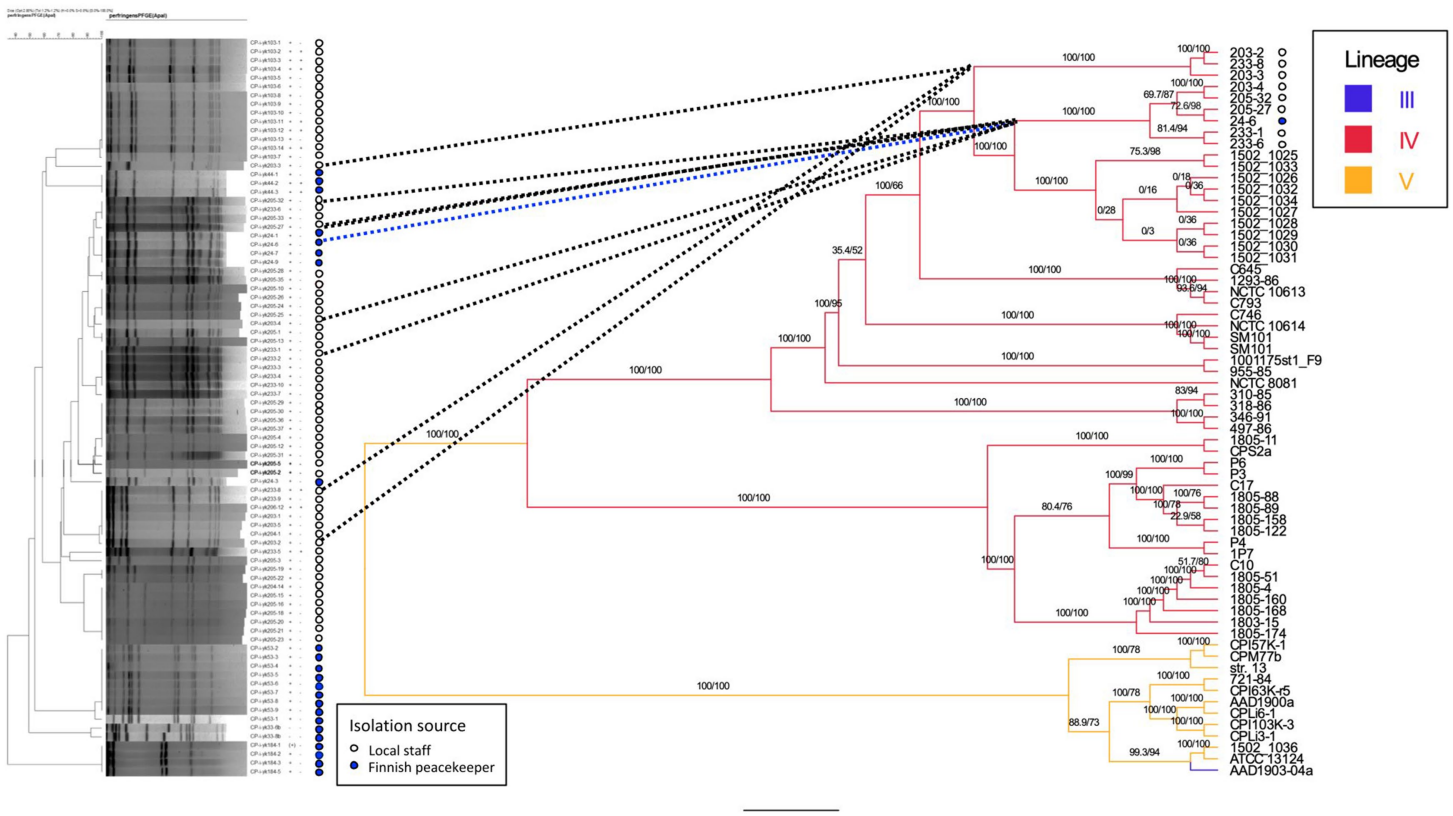
Phylogenetic tree of sequenced strains and previously published strains. Comparison is made between the dendrogram (Dice coefficient with 2%, UPGMA clustering, 84 isolates) of PFGE results (left) and the phylogenetic tree (IQ-TREE 2.3.0, visualized by FigTree v1.4.4., 63 isolates) based on whole-genome sequences (center, right). For the strains isolated in this study, the isolation source (local staff, peacekeeper) is annotated next to the PFGE tree, and bootstrapping values for tree nodes are given. For sequenced genomes and previously published genomes, the genetic lineages (III, IV, and V) of strains are indicated on the right. Nomenclature of strains: CP-i-yk was used as the study ID in the PFGE analysis. The numbers and hyphen (4–6 characters) following the study ID constitute the strain/genome ID. Reference strain = *C. perfringens* strain SM101.

We typed 78 (83%) of 94 *cpe*-positive isolates with PFGE. Isolates from four peacekeepers (samples 33, 44, 53, and 103) displayed four distinct patterns (Figure 1). Isolates from a fifth peacekeeper (sample 24) and four locally employed workers (203, 205, 206, and 233) displayed closely related patterns and included both c-*cpe* and variant isolates. Among this cluster, a subcluster of indistinguishable isolates from the fifth peacekeeper and three local staff members was selected for further analysis.

### 3.3 Sequenced genomes

**Figure 2:**
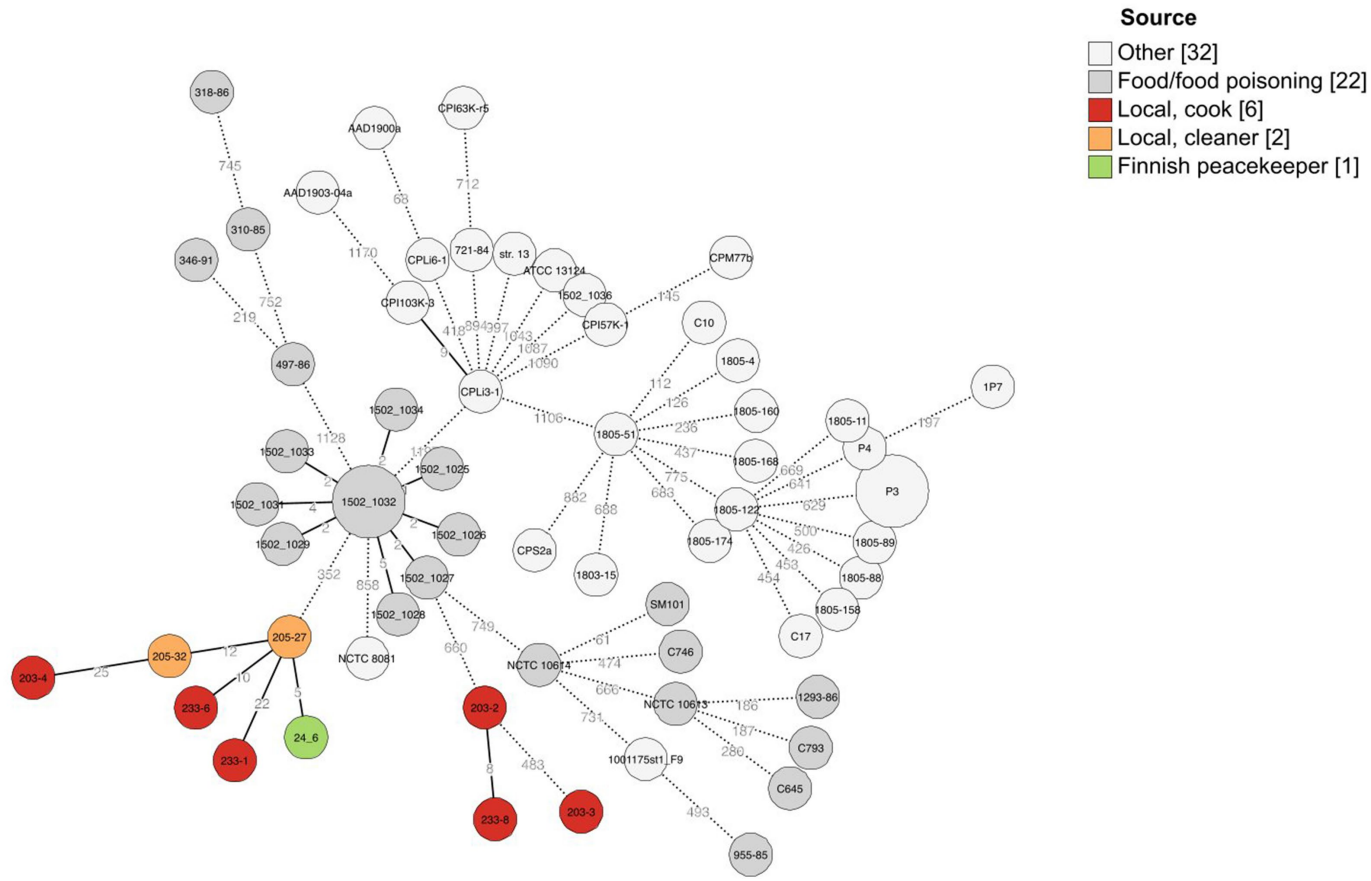
Minimal spanning tree of cgMLST results of 63 *C. perfringens* strains including here sequenced strains (*n* = 9) and previously published *C. perfringens* strains (*n* = 54). A scheme created by Abdel-Glil et al. (2021) was used for allele calling, and 1,236 loci belonging to the core genome were used to create the minimal spanning tree with GrapeTree (MSTreeV2). The isolation sources of strains have been added as a colored annotation, and branches below two allelic differences are collapsed.

Nine isolates representing both c-*cpe* typed isolates and PCR variant *cpe* isolates were sequenced (Table 2). Sequencing revealed that all carried a chromosomally inserted *cpe* and they, despite the varied PCR results and the differences observed in PFGE, belonged to the same phylogenetic lineage IV and phylogenetically clustered together with c-*cpe* food poisoning isolates (Figure 2). Different c-*cpe* groups, 1 and 2, have been recently suggested (Jaakkola et al., 2021). C-*cpe* group 1 strains seem to be equipped for changing pH and acidic, high-temperature environments where iron uptake is competitive, and citrate utilization is beneficial, whereas strains of c-*cpe* group 2 lack these genes and operons. The isolates described here belonged to c-*cpe* group 1, among many well-researched food poisoning strains such as SM101, NCTC 8239, and NCTC 10613.

**Table 2:** Sequenced *C. perfringens* strains, their *cpe* type (chromosomal/plasmidial), initial PCR typing results, genetic lineage, isolation source, and BioSample accession number.

Strain	*cpe* type^a^	Initial PCR typing results	Genetic lineage^b^	Isolation source	BioSample accession number
203–2	Chromosomal	Chromosomal	IV	Cook #1	SAMN35541948
203–3	Chromosomal	Chromosomal	IV	Cook #1	SAMN35541949
203–4	Chromosomal	Variant	IV	Cook #1	SAMN35541950
205–27	Chromosomal	Variant	IV	Cleaner	SAMN35541951
205–32	Chromosomal	Variant	IV	Cleaner	SAMN35541952
233–1	Chromosomal	Variant	IV	Cook #2	SAMN35541953
233–6	Chromosomal	Variant	IV	Cook #2	SAMN35541954
233–8	Chromosomal	Chromosomal	IV	Cook #2	SAMN35541955
24–6	Chromosomal	Variant	IV	Peacekeeper	SAMN35541956

a Based on the sequenced genome.

b Feng et al. (2020) and Jaakkola et al. (2021).

**Figure 3:**
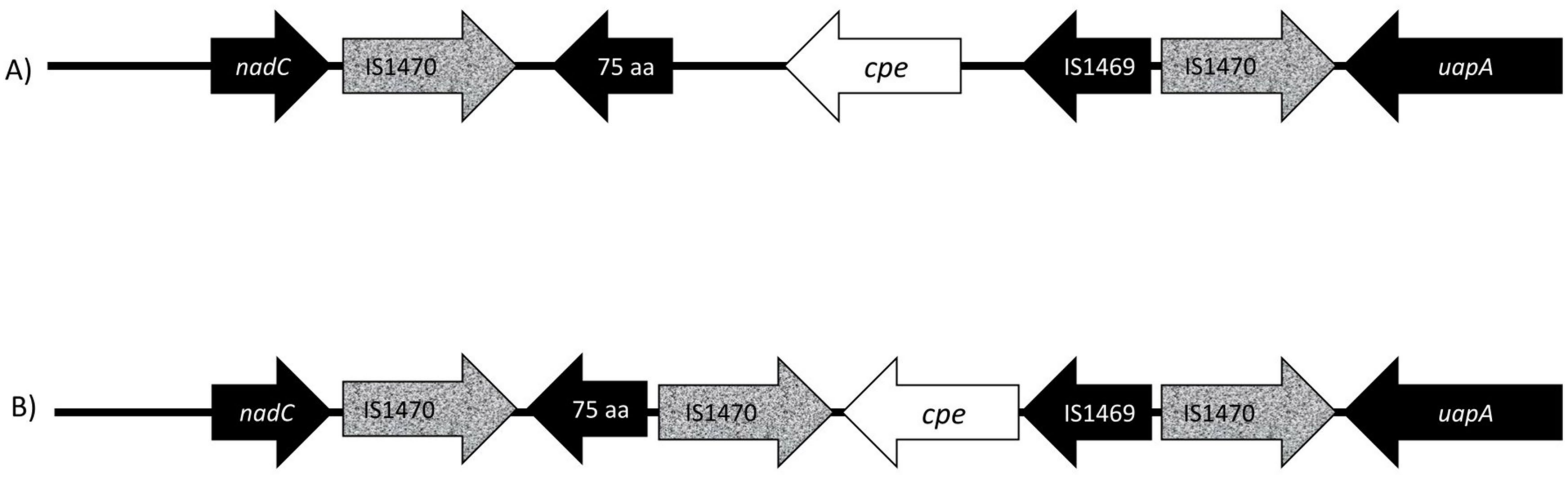
Presentation of gene order on chromosomal insertion sites of enterotoxin gene (*cpe*). **(A)** The well-described and conserved gene order (Brynestad et al., 1997) is commonly utilized for PCR genotyping, and probes target the IS1470 element downstream of the *cpe* gene and the *cpe* gene itself. In this study, six isolates (233–6, 233–1, 205–27, 203–4, 205–32, and 24–6) produced variant PCR results in PCR genotyping and had a new variant *cpe* loci **(B)** with a 1,053 aa IS1470 insertion element directly downstream of the *cpe* gene. The additional IS1470 element was 99% identical to the other, well-described IS1470 in the close vicinity of *the cpe* gene in chromosomal strains.

All sequenced *cpe* genes were highly conserved and encoded a 319 aa enterotoxin sequence. Genome analysis revealed that the six strains in Table 2 with variant *cpe* PCR results (24–6, 203–4, 205–27, 205–32, 233–1, and 233–6) carried an additional IS1470 sequence directly downstream the *cpe* gene (Figure 3), while the genomes typed as chromosomal in PCR (Table 2) had a typical gene order and genetic composition, as described by Miyamoto et al. (2006) around their chromosomally inserted *cpe* gene. For the sake of brevity, these six genes with new gene order for their *cpe* insertion site are called “variant c-*cpe* strains” in this article.

The additional IS1470 element in strains with variant gene order was 99% identical with the other, well-described IS1470 in the close vicinity of the *cpe* gene in chromosomal strains. Further analysis of IS1470 revealed that the variant c-*cpe* strains such as 24–6, 203–4, 205–27, 205–32, 233–6, and 233–1 had also accumulated other additional copies of IS1470 elements (Table 3). Variant c-*cpe* strains carried 58 to 81 copies of IS1470 in their genomes, while the majority of studied chromosomal strains carried 9 to 23 copies of IS1470. P-*cpe* strains carried under 10 copies of IS1470 (0 to 7) (Supplementary Table S2). All c-*cpe* strains carried 1 copy of IS1469 (Supplementary Table S2).

**Table 3:** Comparison of selected genetic features of the c-*cpe* strains sequenced in this study and some well-known *C. perfringens strains*.

*cpe* type	Strain	Lineage	Selected genes and genetic features																		
			N of IS1470	N of IS1469	N of IS1151	*pfoA*	*nanI*	Arc	NanJ	Fhu	FeoAB	Citrate	Cellobiose	Fucose	Ethanolamine	Myo-inositol	Biotin	CspLA	*splB*	SigV	*ssp4* HR
New variant of the c-*cpe* insertion site	203–4	IV	62	1	0	0	0	1	0	1	1	1	0	0	0	0	0	0	0	0	1
	205–27	IV	81	1	0	0	0	1	0	1	1	1	0	0	0	0	0	0	0	0	1
	205–32	IV	57	1	0	0	0	1	0	1	1	1	0	0	0	0	0	0	0	0	1
	233–1	IV	65	1	0	0	0	1	0	1	1	1	0	0	0	0	0	0	0	0	1
	233–6	IV	58	1	0	0	0	1	0	1	1	1	0	0	0	0	0	0	0	0	1
	24–6	IV	58	1	0	0	0	1	0	1	1	1	0	0	0	0	0	0	0	0	1
The known c-*cpe* insertion site	203–2	IV	23	1	0	0	0	0	0	1	0	1	1	0	0	0	0	0	1	0	1
	203–3	IV	31	1	0	0	0	0	0	1	0	1	1	0	0	0	0	0	1	0	1
	233–8	IV	23	1	0	0	0	0	0	1	0	1	1	0	0	0	0	0	1	0	1
	NCTC8081	IV	49	0	2	0	0	0	1	1	1	0	0	0	0	0	0	0	1	0	1
	NCTC8239	IV	23	1	0	0	0	1	0	1	1	1	1	0	0	0	0	0	0	0	1
	SM101	IV	16	1	0	0	0	1	0	1	1	1	1	0	0	0	0	0	0	1	1
	NCTC10240	IV	9	1	0	0	0	0	1	0	0	0	1	1	0	0	0	0	0	0	0
Plasmidial *cpe*	CPE str. F4969	V	0	2	0	1	1	1	1	1	1	0	0	1	1	1	1	1	1	1	0
	AAD1903a	III	0	1	1	1	1	1	1	1	1	1	0	0	1	1	1	1	1	1	0
*cpe-*negative	Str 13	V	0	0	0	1	1	1	1	1	0	1	0	0	1	1	1	1	1	1	0

*Cpe* type (New variant of the c-*cpe* insertion site/The known c-*cpe* insertion site/Plasmidial *cpe*/*cpe*-negative), the genetic lineage, number of IS elements (>80% identity, >80% length), and presence (1) and absence (0) of selected genes and genetic features [Arc = arginine deiminase pathway, Fhu = ferrichrome uptake system, FeoAB = iron uptake system, Citrate = citrate metabolism, Cellobiose = cellobiose metabolism operon, Fucose = fucose metabolism operon, Ethanolamine = ethanolamine utilization operon, Myo-inositol = myo-inositol utilization operon, Biotin = biotin biosynthesis, SplB = spore photoproduct lyase, SigV = sigma V, Ssp4 HR = gene allele with Asp at residue 36 and Asn at residue 72 associated with spore heat resistance (Li and McClane, 2008)] in sequenced *C. perfringens* strains. All IS1470 hits were located within the chromosome. Complete version of this table is available in Supplementary Table S2.

The traditional and variant c-*cpe* strains sequenced in this study had slight differences when selected genetic features were compared (Table 3). The traditional c-*cpe* strains lacked the arginine deiminase pathway Arc and the iron uptake system FeoAB, which were present in variant c-*cpe* strains. The traditional c-*cpe* strains sequenced in this study carried cellobiose metabolism operon and spore photoproduct lyase SplB, which were absent in the variant c-*cpe* strains. Sequenced strains were subjected to cgMLST analysis to characterize the strains and shed light on their epidemiological context. Analyzed *C. perfringens* strains (Supplementary Table S1) shared 1,236 genes in their core genome. Strains 24–6, 203–4, 205–27, 205–32, 233–1, and 233–6 formed a clade and shared highly similar cgMLST gene profiles (5 to 25 differences), suggesting genetic relatedness and a recent shared origin (Figure 1). These strains had been isolated from a peacekeeper (24–6), two local kitchen staff members (Cook #1203: 203–4 and Cook #2233: 233–1 and 233–6), and a local cleaner (205–27 and 205–32). Strains 203–2, 203–3, and 233–8 shared another clade with a genetic difference of 8 between 203–2 and 233–8 (Figure 2).

## 4 Discussion

In the present study, we found *C. perfringens* type F strains in the stool samples of peacekeepers and locally employed workers. Among the samples from locally employed workers and peacekeepers, 47 and 17%, respectively, tested positive for *cpe*-carrying *C. perfringens*. Altogether, 94 c-*cpe* strains were isolated from 12 people, including clinically healthy individuals (the locally employed workers and some peacekeepers) and those who were ill or recovering from intestinal disease (some peacekeepers). The isolation of c-*cpe* strains from multiple people suggests that there may have been one or more *C. perfringens* food poisoning outbreaks among peacekeepers with symptoms of disease as c-*cpe* strains are known to be a common cause of food poisoning (Lindström et al., 2011). However, since the sampling was conducted in October and the symptoms of the peacekeepers had occurred between June and October, CPE or large numbers of type F isolates were not found in the feces of affected people, and we could not definitively identify these symptoms being caused by *C. perfringens* type F. The local workers were not included in the epidemiological questionnaire or systematically interviewed, so it is unclear whether they had some symptoms of intestinal disease at the same time period as the peacekeepers. Interestingly, we obtained only c-*cpe* isolates, especially among locally employed workers. P-*cpe* strains are often present in the normal intestinal microbiota of many healthy people (Heikinheimo et al., 2006; Carman et al., 2008). Noteworthy, the isolation of *C. perfringens* strains was not successful from all *cpe*-positive samples, and whether p-*cpe* or c-*cpe* strains were present in these samples remains unknown.

In the present study, four peacekeepers had individual c-*cpe* strains according to the PFGE results. Those c-*cpe* strains were not found in anyone else. Based on the questionnaire, at least two of these peacekeepers had the symptoms of intestinal disease 1–2 months earlier, but not at the time of sampling. Moreover, one peacekeeper shared the same c-*cpe* strain with two local cooks and one local cleaner. According to the questionnaire, this peacekeeper had symptoms of intestinal disease 3 weeks earlier but not at the time of sampling. This may indicate that the peacekeepers had become carriers of the c-*cpe* strains after food poisoning.

While there is no clear evidence that c-*cpe* strains have been the cause of symptoms observed among the peacekeepers, the fact that we found genetically similar *C. perfringens* strains in samples from the locally employed workers and peacekeepers suggests that transmission of these strains has occurred among camp crew. The transmission has most likely been foodborne as this is the most common route of transmission for intestinal bacteria (Browne et al., 2017). This is further supported by the presence of the same strains in the kitchen staff. The high number of c-*cpe C. perfringens* among the local workers may even suggest humans as a reservoir of the c*-cpe* strains, and the persistence of c-*cpe* in peacekeepers several months after symptoms may even suggest that humans might become at least a transient reservoir of c-*cpe* strains in some cases. This subject needs to be further elucidated for better prevention of epidemics in the future. Reservoirs of the c-*cpe* strains are unclear, but humans as reservoirs have also been suggested previously (Heikinheimo et al., 2006).

In a previous study (Kiu et al., 2019), genetically highly similar p-*cpe C. perfringens* strains were associated with nine distinct care-home-associated outbreaks throughout a 5-year interval. Kiu et al. (2019) suggested that there was a common source linked to these outbreaks or transmission over time and space. Previous studies (Lahti et al., 2012; Jaakkola et al., 2021) have suggested that p-*cpe* strains and some c-*cpe* strains inhabit human intestines, so humans as carriers of food poisoning strains over time and space are possible. Since peacekeepers had symptoms evenly distributed during summer and autumn in 2004, it is possible that there had been a persistent food poisoning outbreak caused by the c-*cpe* strains carried by the kitchen workers.

An alternative explanation is that contaminated food ingredients have introduced the *C. perfringens* strains to the camp, where camp conditions may have facilitated the transmission of c-*cpe* strains among food consumers. C-*cpe* strains are known to enter the food chain and have been identified as the sole or predominant *cpe*-positive strains in retail meats from both the United States (Wen and McClane, 2004) and Turkey (Yibar et al., 2018). However, it is unclear how the c-*cpe* strains end up in the food chain, from food handlers or other sources. In the present study, the camp’s food premises were adequate when inspected, and the UN procured food from international operators known for their high standards of food safety.

In the present study, the higher proportion of the c-*cpe C. perfringens* among the local workers than among the peacekeepers may also indicate a specific local source of c-*cpe C. perfringens*. The high numbers of these pathogenic isolates in the stool of one local cleaner (32 isolates) and a local cook (10 isolates) support this. The exposure to local foods outside the camp may have increased the risk of *C. perfringens* food poisoning.

The cgMLST scheme for *C. perfringens* has been used to type strains in several studies, and allele differences between these variable species are usually relatively large, with differences ranging from 200 to 1,000 alleles between strains (Abdel-Glil et al., 2021; Jaakkola et al., 2021). The allele differences between these sequenced strains varied between 5 and 25, and they formed a distinct cluster, supported by a separate clade in phylogenetic analysis (Figure 2). The differences between epidemiologically linked isolates are usually below 10 alleles in bacterial species (Schürch et al., 2018), but the cluster thresholds are dependent on the sequencing method and bacterial species, and larger cluster thresholds have been suggested for *C. perfringens* and its highly variable genome (Abdel-Glil et al., 2021). We suggested that the isolation of strains with less than 50 allele differences from a diverse group of people working on the same campsite suggests recent genetic relatedness, and considering the associated symptoms of gastrointestinal disease, there is a possible persistent outbreak among the residents at the camp. It is also possible that the chosen sequencing method has introduced some observed differences between the isolates.

The *cpe* insertion sites in the *C. perfringens* are well-described and conserved; therefore, they are widely used for strain typing. In our PCR typing, 45 of 94 c-*cpe* isolates were variants, and the sequencing revealed that the sequenced strains with variant results had an additional IS1470 element inserted directly downstream of the *cpe* gene. Variation in *cpe* loci arrangements has been previously reported by Abdelrahim et al. (2019), but this is the first time a *cpe* loci arrangement has been reported to affect PCR typing. Furthermore, the additional IS1470 element within *cpe* loci observed here has not been previously described.

The PCR genotyping probes in the *C. perfringens* assay target the IS1470 element downstream of the *cpe* gene and the *cpe* gene itself, and the presence of an additional IS1470 element downstream of the *cpe* gene impaired the standardized genotyping PCR assay. These variant c-*cpe* strains could be misidentified as strains with unknown *cpe* locations. Due to the variant c-*cpe,* the genotyping PCR primers need to be redesigned to detect reliably the variant c-*cpe* as well.

The traditional and variant c-*cpe* strains sequenced in this study all belonged to c-*cpe* group 1, which tolerate changing pH and acidic environments (Jaakkola et al., 2021). Interestingly, the variant c-*cpe* strains and traditional c-*cpe* strains had slight differences when selected genetic features were compared (Table 3). The variant c-*cpe* strains carried the arginine deiminase pathway Arc and the iron uptake system FeoAB, which were absent in traditional c-*cpe* strains and lacked the cellobiose metabolism operon, which was present in the traditional c-*cpe* strains. These possible differences in the metabolism between the c-*cpe* types may indicate that the variant c-*cpe* strains are better equipped for harsh conditions.

The variant c-*cpe* strains with an additional IS1470 element downstream of the *cpe* gene had a higher number of IS1470 elements in their entire genome compared to other *C. perfringens* strains. IS1470 element has been reported on chromosomes, never plasmids, and generally in moderate numbers (0–10 copies) (Brynestad et al., 1994). However, IS1470-like sequences are common on *C. perfringens* plasmids (Miyamoto et al., 2004). IS elements together with other transposable elements are important mutagenic agents enabling the host to adapt to new environmental challenges and colonize new niches. IS expansion has been linked to genome rearrangements, genome size reduction, and gene inactivation characteristic of the emergence of pathogenic strains (Parkhill et al., 2003; Siguier et al., 2006; Vandecraen et al., 2017), with famous examples of *Yersinia pseudotuberculosis* and *Yersinia pestis* (Parkhill et al., 2001; Chain et al., 2004), *Bordetella bronchiseptica*, and *Bordetella pertussis* (Parkhill et al., 2003).

In *C. perfringens*, the expansion of IS1470 elements has previously been reported in Darmbrand strain NCTC 8081 (Ma et al., 2012), and in other bacteria, the expansion of IS elements has been associated with an increase in virulence (Parkhill et al., 2003; Siguier et al., 2006). The relevance of IS1470 expansion for these Eritrean strains remains unknown, but the impact of IS1470 expansion on *C. perfringens* gene expression and host adaptation would be interesting topics for further research.

## 5 Conclusion

Our results suggest that persistent food poisoning outbreaks caused by *C. perfringens* type F strains can occur and that humans are a likely reservoir and carrier for enteropathogenic *C. perfringens*. We also conclude that the occurrence of additional IS1470 elements in chromosomal *cpe*-carrying *C. perfringens* strains can impair the PCR typing, resulting in false-negative typing of these strains.

We present six new c-*cpe C. perfringens* genomes featuring an additional IS1470 element at the *cpe* insertion site and describe the organization of this new variant of the *cpe* locus. In addition, these variant c-*cpe* strains carry 58–81 copies of IS1470 in their genomes instead of 9–23 copies in previously described chromosomal *cpe* strains.

Our study contributes to the expansion of the pool of c-*cpe* strains by introducing Eritrean strains marking the first reported instances of c-*cpe* strains originating from Eastern Africa.

## Data Availability

The datasets presented in this study can be found in online repositories. The names of the repository/repositories and accession number(s) can be found in the article/Supplementary material.

## Footnotes

^1^ https://www.bv-brc.org/
